# 

**Published:** 1882-05

**Authors:** 


WHOLE NUMBER, CCXXXVIII.

MAY, 1882.

NUMBER 10, VOLUME XXI.

S.G.O.L. INDEXED

### THE BUFFALO Medical and Surgical Journal.

EDITORS:

THOS. LOTHROP, M. D., A. R. DAVIDSON, M. D.,

P. W. VAN PEYMA, M. D., LUCIEN HOWE, M. D.,

HERMAN MYNTER, M. D.

BUFFALO:

PUBLISHED BY THE MEDICAL JOURNAL ASSOCIATION.

**Read the Notice of this Journal among the Advertisements.**

Entered at the Post-Office at Buffalo, N. Y., as Second-Class Mail Matter.

